# NADPH oxidase is the major source of placental superoxide in early pregnancy: association with MAPK pathway activation

**DOI:** 10.1038/s41598-019-50417-4

**Published:** 2019-09-27

**Authors:** Isabelle Hernandez, Thierry Fournier, Audrey Chissey, Patrice Therond, Abdel Slama, Jean-Louis Beaudeux, Amal Zerrad-Saadi

**Affiliations:** 1 INSERM UMR-S 1139: Physiopathologie et Pharmacotoxicologie Placentaire Humaine/ Microbiote Pré et Postnatal, Paris, 75006 France; 2 Université de Paris, Université Paris Descartes, Paris, 75006 France; 3 Service de Biochimie, Hôpital Universitaire Kremlin Bicêtre, Hôpitaux Universitaire Paris Sud, Assistance Publique des Hôpitaux de Paris, Le Kremlin-Bicêtre, 94275 France; 4 EA7537, Université Paris Sud et Université Paris Saclay, Chatenay-Malabry, 92290 France; 5 Service de Biochimie Générale, Hôpital Universitaire Necker-Enfants malades, Assistance Publique des Hôpitaux de Paris, Paris, 75015 France; 6 Fondation Premup, Paris, 75005 France

**Keywords:** Pathogenesis, Stress signalling, Embryology

## Abstract

First-trimester placenta (<10 gestational weeks (GW)) develops in a low oxygen environment (≈2%). Early oxygen exposure can cause oxidative damage leading to pregnancy disorders. The aim of this work was to determine the major sources of placental superoxide during early pregnancy - more specifically before 10 GW - and to study redox adaptation to increased oxygen pressure after 12 GW. Our results show that NADPH oxidase (Nox) is the main source of superoxide in first-trimester chorionic villi. Its activity is higher before 10 GW and concomitant with the location on the syncytiotrophoblast apical pole of p47phox, the Nox organizer subunit. After the increase in pO_2_ pressure (12–14 GW), the activities of the antioxidant enzymes SOD1, catalase and GPX1 are increased. The redox-sensitive MAPK pathways show increased phosphorylated-p38 expression, but no variation in the phosphorylation of stress-activated protein kinase/c-Jun NH2-terminal kinase (SAPK/JNK) during first trimester, suggesting a physiological redox adaptation, whilst ERK1/2 phosphorylation is higher after 12 GW. Nox is the major superoxide source in early pregnancy (<10 GW). Increased superoxide production at 7–9 GW is associated with p38 MAPK pathway activation, suggesting that it is involved in physiological placental function and healthy early development of the placenta, through MAPK pathways.

## Introduction

During pregnancy, the chorionic villus, which represents the essential structural and functional component of the human placenta, undergoes drastic changes. It first develops in a low-oxygen environment throughout the first trimester^1^ due to the obstruction of uterine arteries by the invasive trophoblastic cells; the oxygen pressure is about 20 mmHg in the intervillous space. During the physiological remodeling of arteries, trophoblastic plugs disappear, allowing unrestricted flow of maternal oxygenated blood to the intervillous space. The consequence is an important modification of the trophoblast environment, including an increase in oxygen level from about 20 mmHg to about 60 mmHg^2–4^. The syncytiotrophoblast (ST), which covers the entire surface of the villus, is in direct contact with a maternal blood exudate during the early first trimester of pregnancy and with maternal whole blood from 12 gestational weeks (GW). The ST plays a major role in fetomaternal exchanges throughout pregnancy and in synthesis of steroid and peptide hormones required for pregnancy, fetal growth and development. The ST represents the exchange and endocrine tissue of the human placenta^5^ for which the redox status during this critical low-oxygen to high-oxygen switch remains to be clarified^3^. Although a defect in redox status in the early placenta has already been attributed to the pathogenesis of preeclampsia and intrauterine growth restriction (IUGR)^6–8^, the mechanisms have not been clearly identified.

Redox adaptation of the cell is involved in the balance between pro- and antioxidant species in maintaining a low basal level of reactive oxygen species (ROS). A major physiological source of ROS is the NADPH oxidase (Nox) family. Nox are a family of isoenzymes composed of transmembrane hemoproteins and electron transporters. Their sole function is to catalyze the reduction of molecular oxygen to superoxide anion O_2_•^−^. The Nox family is composed of seven members including Nox1, Nox2 (also called gp91phox), Nox3, Nox4, Nox5 and DUOX1/2. Nox2 is highly conserved among species^9^, which highlights its essential function. It is the most described isoform and is expressed in phagocytic cells. Nox has been described in several cell types, especially in the neutrophils where production of superoxide anion is involved in phagocytosis, but few studies have explored the isoform of Nox in human placenta. The identified Nox placental isoform appears to be different from the neutrophil isoform, being constituted of 58 kDa and 33 kDa subunits^10^. It has been shown that Nox is the main source of superoxide production in 10–12 GW and term placenta^11^. There are no data about the placental source of superoxide production before 10 GW in the absence of oxygenated maternal blood in the intervillous space.

Mitogen-activated protein kinases (MAPKs) are involved in signal transduction in cells resulting from extracellular stimuli. MAPK pathways work as an evolutionarily conserved phosphorylation cascade: an extracellular signal will trigger GTP-binding protein of the Ras/Rho family^12^ or STE20 protein^13^, which in turn phosphorylates MAPKK kinase or MEK kinase (MAPKKK or MEKK), then MAPK kinase (MAPKK, MKK or MEK) and finally MAPK^14^. There are currently four MAPKs: p38 (α/β/δ/γ), extracellular signal-related kinases (ERK 1/2), Jun amino-terminal kinases (JNK 1/2/3) and ERK5. Each MAPK is activated by a specific MAPKK^14^, which improves the specificity of the intracellular response. ERK1/2 is involved in cell proliferation, whereas p38 is often reported to be involved in pro-inflammatory processes and apoptosis^12^. As redox-sensitive pathways, MAPK may be activated by Nox: in endothelial cells, Nox1, Nox2 and Nox4 isoforms are involved in inflammatory response and cytokine expression, triggered by angiotensin II treatment, through different MAPK pathways activation (p38, ERK ½ and SAPK/JNK)^15^. In placenta, these pathways seems to be involved in trophoblast differentiation and proliferation^16–19^.

The aim of this work was to study the major sources of superoxide during the early stages of pregnancy and, for the first time, more specifically before 10 GW, when chorionic villi are exposed to relative hypoxia. The second objective was to study the adaptation of Nox activity throughout the first trimester. We also studied redox adaptation of the human placenta to the increase in partial oxygen pressure during the early environmental transition. Hence, we measured superoxide production with or without specific inhibitors of superoxide producers (Nox, mitochondrial respiratory chain and endothelial nitric oxide synthase (eNOS)) and then studied the activity and location of the main antioxidant enzymes before and after the critical low-oxygen (7–9 GW) to high-oxygen (12–14 GW) switch, as well as the phosphorylated expression of MAPK proteins, which has never been done before.

## Materials and Methods

### Ethical statement

First-trimester placental tissues used for this study were obtained after the patients gave written informed consent at the Cochin Port-Royal and Institut Mutualiste Montsouris hospital maternity units. All experiments were performed in accordance with the relevant guidelines and regulations. The protocol was approved by the institutional review board “Comité de Protection des Personnes 2015-mai-13909”.

### Materials

Hank’s balanced salt solution (HBSS, ref: 14175-053) and phosphate buffer saline (PBS, ref: 14190-094) were from Thermo Fisher Scientific (Waltham, MA, USA), as were the protein assay kit Micro BCA™ Assay Kit (ref: 23235), Triton X-100 (ref: 11891445) and Tissue Protein Extraction Reagent T-PER™ (ref: 78510). Protease inhibitor cocktail (ref 539131) and phosphatase inhibitor cocktail (ref: 524629) were from VWR (Radnor, PA, USA).

For enzymatic activity assays, potassium dihydrogen phosphate (KH_2_PO_4,_ ref: 1.05104) was from Merck Millipore (Burlington, MA, USA), glycol ether diamine tetraacetic acid (EGTA, ref: E-4378) was from Sigma Aldrich (Saint-Louis, MO, USA) and sucrose (ref: 35579) from SERVA Electrophoresis GmbH (Heidelberg, DE). Lucigenin (ref: 14872) was from Cayman (Ann Arbor, MI, USA) and nicotinamide adenine dinucleotide phosphate H (NADPH, ref: N7505), diphenyleneiodonium (DPI, ref: D2926), N(ω)-nitro-L-arginine methyl ester (L-NAME, ref: N5751), rotenone (ref: R8875), and the Xanthine Oxidase Assay Kit (ref: MAK078) were from Sigma Aldrich (Saint-Louis, MO, USA). Ransod (ref: SD125) and Ransel (ref: RS505) kits were from Randox Laboratory (Crumlin, Northern Ireland, UK).

For immunolocalization studies, paraformaldehyde (PFA, ref: 15710) was from Euromedex (Souffelweyersheim, Bas-Rhin, FR) and agarose (ref: 444153 H) from VWR (Radnor, PA, USA). Goat serum (ref: 005-000-121) and human IgG (ref: 009-000-003) were from Jackson ImmunoRechearch (Ely, UK). Alexa Fluor 488 and 546, To-pro®-3 iodide (642/661, ref: 10710194), and DyLight Fluor-conjugated antibody (600 or 800 conjugate) were from Life Technologies (Carlsbad, CA, USA). Bovine serum albumin IgG-free (BSA, ref: 011-000-162) was from Interchim (San Diego, CA, USA) and VectaFluor R.T.U DyLight® 488 Anti-Rabbit IgG system (ref: DK-1488) from Cliniscience (Nanterre, Hauts-de-Seine, FR). Anti-keratin 7 rabbit polyclonal antibody (ref: SAB4501652), Phalloidin-Atto 550 (ref: 19083), and DAPI (ref: D9542) were from Sigma Aldrich (St. Louis, MO, USA). Monoclonal mouse anti-human cytokeratin 7 (clone OV-TL 12/30, ref: M7018) was from Dako (Agilent Technologies (Santa Clara, CA, USA)). Catalase XP^®^ Rabbit monoclonal antibody (ref: D4P7B), SOD1 mouse monoclonal antibody (ref: 71G8) anti-phospho-SAPK/JNK (Thr183/Tyr185) mouse monoclonal antibody (ref: 9255), anti-SAPK/JNK rabbit polyclonal antibody (ref: 9252), anti-phospho-p44/42 MAPK (ERK1/2) (Thr202/Tyr204) XP^®^ rabbit monoclonal antibody (ref: 4370), anti-p44/42 MAPK (ERK1/2) rabbit monoclonal antibody (ref: 4695), anti-phospho-p38 MAPK (Thr180/Tyr182) XP^®^ rabbit monoclonal antibody (ref: 4511), and anti-p38 MAPK XP^®^ rabbit monoclonal antibody (ref: 8690) were from Cell Signaling Technology (Danvers, MA, USA), and anti-p47phox rabbit polyclonal antibody (ref: 07-001) was from Merck Millipore (Burlington, MA, USA).

### Collection of chorionic villi

Chorionic villi were collected from first-trimester placenta (7–9 and 12–14 GW), washed in HBSS 1X at 37 °C, dissected free of membranes and centrifuged for 10 min at 200 g. The supernatant was removed and the tissue was quickly frozen in approximately 60 mg/65 mm^3^ samples, and then stored at −80 °C.

### Enzyme assays

NADPH oxidase activity assay was adapted from Raijmakers *et al*.^11^: frozen villi were thawed in a cold buffer solution (5% w/v; 50 mmol/L KH_2_PO_4_, 1 mmol/L EGTA, 150 mmol/L sucrose, pH 7.4) containing 0.1% protease inhibitor cocktail, then homogenized with an Ultra-Turrax® (GmbH & Co. KG, Esslingen, DE). Each well (total volume in buffer: 90 µL) was filled with the sample homogenate (18 µL), 5 µM lucigenin and 400 µM NADPH. The source of superoxide was determined using specific inhibitors: DPI (targeting NADPH oxidase: final concentration 10 µmol/L), L-NAME (targeting NO synthase: final concentration 100 µmol/L) and rotenone (targeting the mitochondrial respiratory chain: final concentration 20 µmol/L). The inhibitor assays were performed on the same sample in order to be compared with control and repeated on different placentas for each group. After 10 min of dark adaptation, luminescence (arbitrary light units (ALU)) was monitored for 30 min at 37 °C using a microplate reader (Enspire® 2300 Multilabel Reader® - PerkinElmer (Waltham, MA, USA)). The assays were performed in triplicate. The protein concentration was determined using the Micro BCA™ Assay Kit. For each assay, the area under the curve (AUC) was calculated and considered as the superoxide production.

Superoxide dismutase 1 (SOD1) activity (Cu/Zn-SOD; EC 1.15.1.1) was measured with a commercially available Ransod kit based on the method developed by McCord and Fridovich^20^. Glutathione peroxidase 1 (GPX1; EC 1.11.1.9) activity was measured with a commercially available Ransel kit using a method based on that developed by Paglia and Valentine^21^. Catalase (CAT; EC 1.11.1.6) activity was measured with a spectrophotometric method as previously described by Johansson and Borg^22^. Xanthine oxidase (EC 1.17.3.2) activity was assayed using the Xanthine Oxidase Assay kit according to the manufacturer’s instructions. These activities were normalized to protein concentration, determined using the Micro BCA™ Assay Kit.

Respiratory chain activities of complex II (succinate–ubiquinone oxidoreductase; EC 1.3.5.1), complex III (ubiquinone–cytochrome c oxidoreductase; EC 1.10.2.2), complex IV (COX; EC 1.9.3.1) and citrate synthase (EC 2.3.3.1) activity were measured in a Cary 50 Spectrophotometer (Agilent Technologies (Santa Clara, CA, USA))^23^. The assays were performed on villi homogenates, and their activities normalized to protein concentration.

### Immunolocalization studies

Immunohistochemistry was performed on human first-trimester placenta. Fresh villi were fixed for 4 hours in 4% PFA at 4 °C, then in 1% PFA at 4 °C overnight, washed in PBS 1×, and embedded in 4% (w/v) agarose. Sections (80–120 µm) were permeabilized with 0.5% Triton X-100 and blocked in 3% BSA IgG-free, 5% goat serum, human IgG (final concentration: 12.5 µg/mL), 0.01% Triton X100 and incubated with anti-catalase (0.25 µg/mL) or anti-SOD1 (4 µg/mL) and anti-cytokeratin 7 (mouse: 1.3 µg/mL; rabbit: 2 µg/mL). Sections were incubated with the appropriate fluorochrome-conjugated secondary antibody (1:500 Alexa Fluor 488 or 546). Nuclei were stained with 1:500 To-pro®-3 iodide. For p47phox location, sections were incubated with anti-p47phox (50 µg/mL) and 1:200 Phalloidin–Atto 550. To amplify the p47phox signal, the VectaFluor R.T.U. DyLight^®^ 488 Anti-Rabbit IgG system was used. Nuclei were stained with DAPI (0.2 µg/mL). Acquisitions were made with a Leica® SP2 confocal microscope and analyzed with ImageJ software (Bethesda, MD, USA).

### Protein extraction and immunoblot analysis

Protein extraction from first-trimester villi was performed using extract buffer T-PER™ containing 0.01%, protease inhibitor cocktail and 0.02% phosphatase inhibitor cocktail. Villi were sonicated for 20 s and centrifuged for 10 min at 10,000 rpm at 4 °C. Supernatants were collected and stored at −80 °C.

Protein concentrations were determined using the Micro BCA™ Assay Kit following the manufacturer’s instructions. Plate reading was performed using an Enspire® spectrophotometer.

After boiling for 5 min, protein extracts were resolved by 10% SDS-PAGE and immunoblotted with anti-p47phox (0.035 µg/mL), anti-phospho-p38 and total p38 (1:1000), anti-phospho-ERK1/2 (1:2000), anti-ERK1/2 (1:1000), anti-phospho-SAPK/JNK (1:2000) or anti-SAPK/JNK (1:1000) antibodies. Blots were revealed using the Odyssey infrared imaging system and analyzed with Odyssey application software v3.0 (Li-Cor Bioscience, Lincoln, NE, USA) after incubation with appropriate DyLight Fluor-conjugated secondary antibody (680 or 800 conjugate).

### Statistical analysis

All data were analyzed with GraphPad Prism software (La Jolla, CA, USA) using the Mann-Whitney test for two group comparisons. For more than two groups (superoxide production assay), the Friedman test was used followed by Dunn’s multiple comparison post hoc test if significant. An unpaired t-test was used to compare age of maternity and gestational age between the two groups, and Fisher’s exact test was used to study the percentage of smokers in the two groups. A *p-*value lower than 0.05 was considered statistically significant, with p < 0.05 and p < 0.01 represented as * or **, respectively. Graphical representations show experimental results with median ± interquartile range.

## Results

### Studied population

All placentas were obtained from singleton pregnancies after voluntary abortion; none were from an assisted reproductive technology (ART) procedure. Gestational age was determined using ultrasonography embryo crown-rump length measurement. These elective terminations of pregnancy were conducted in cases of a presumably normal fetus. When miscarriages or fetal abnormality were diagnosed before the surgical intervention, placentas were not included in the study. No patient was addressed for elective termination of pregnancy in a context of fetal genetic disease or morphologic anomaly.

There were no between-group differences in mean maternal age at termination of pregnancy (26.1 years [±5.5 years] for the 7–9 GW group and 26.5 years [±6.8 years] for the 12–14 GW group) or in the percentage of smokers in our population (35% at 7–9 GW and 50% at 12–14 GW). Mean gestational ages were 8 GW and 6 days (±4 days) in the 7–9 GW group and 13 GW (±7 days) in the 12–14 GW group.

### NADPH oxidase is the main source of superoxide in first-trimester placenta

**Figure 1:**
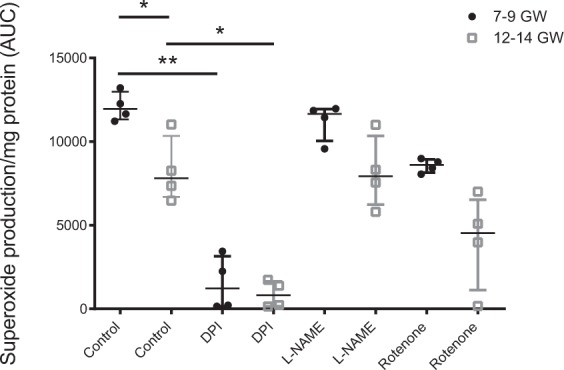
Superoxide production in 7–9 vs. 12–14 GW chorionic villi (AUC: area under the curve, n = 4 different placentas) was measured, with or without inhibitors of superoxide producers (molecule/target: DPI/NADPH oxidase; L-NAME/eNOS; Rotenone/mitochondrial respiratory chain). Results are represented as median ± interquartile range. *p < 0.05, **p < 0.01. DPI: diphenyleneiodonium, L-NAME: N(ω)-nitro-L-arginine methyl ester, eNOS: endothelial nitric oxide synthase.

Because Nox is a major source of ROS in several cell types, placental Nox superoxide production was measured using lucigenin assay with or without inhibitors, selected for their specific inhibition capacity against sources of superoxide O_2_^.−^. The results showed almost complete inhibition of superoxide production on addition of Nox inhibitor (DPI: diphenyleneiodonium), regardless of gestational age (Fig. 1). The addition of L-NAME (nitric oxide synthase inhibitor) or rotenone (mitochondrial respiratory chain inhibitor) had no significant impact on superoxide production, confirming the specificity of the method in measuring Nox activity in first-trimester placenta (Fig. 1). When Nox activity was measured in untreated chorionic villi (control), the results showed a 30% increase in Nox activity in the early stage (7–9 GW) of first-trimester pregnancy (Fig. 1).

**Figure 2:**
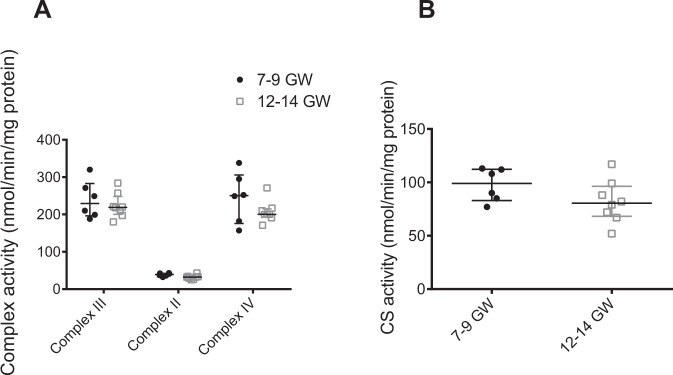
(**A**) Activities of the different complexes of the mitochondrial respiratory chain in 7–9 vs. 12–14 GW chorionic villi were measured (7–9 GW: n = 6, 12–14 GW: n = 8 different placentas). (**B**) The citrate synthase (CS) activity, reflecting the mitochondrial mass, was also measured (7–9 GW: n = 6, 12–14 GW: n = 8 different placentas). Data are represented as median ± interquartile range.

Mitochondrial production of superoxide is often described as a major source of ROS. In order to confirm that the mitochondrial respiratory chain is not involved in superoxide production during the first trimester of pregnancy, the activities of complexes II, III, and IV of the mitochondrial respiratory chain in both 7–9 and 12–14 GW chorionic villi were measured (Fig. 2A). The citrate synthase activity (Fig. 2B), reflecting the mitochondrial mass, was also measured in the two groups. No difference was found in the activity of any complexes or citrate synthase (Fig. 2A,B), and confirms that the variation of superoxide production in first-trimester placenta does not involve mitochondria.

Finally, activity of xanthine oxidase, which is also described as a source of superoxide in cells, was measured in first-trimester chorionic villi and remains undetectable. The addition of 100 μM allopurinol (xanthine oxidase inhibitor) did not change superoxide production, regardless of gestational age (data not shown).

### NADPH oxidase activity increases in very early placenta (<10 GW) and is related to the location of p47phox organizer subunit

**Figure 3:**
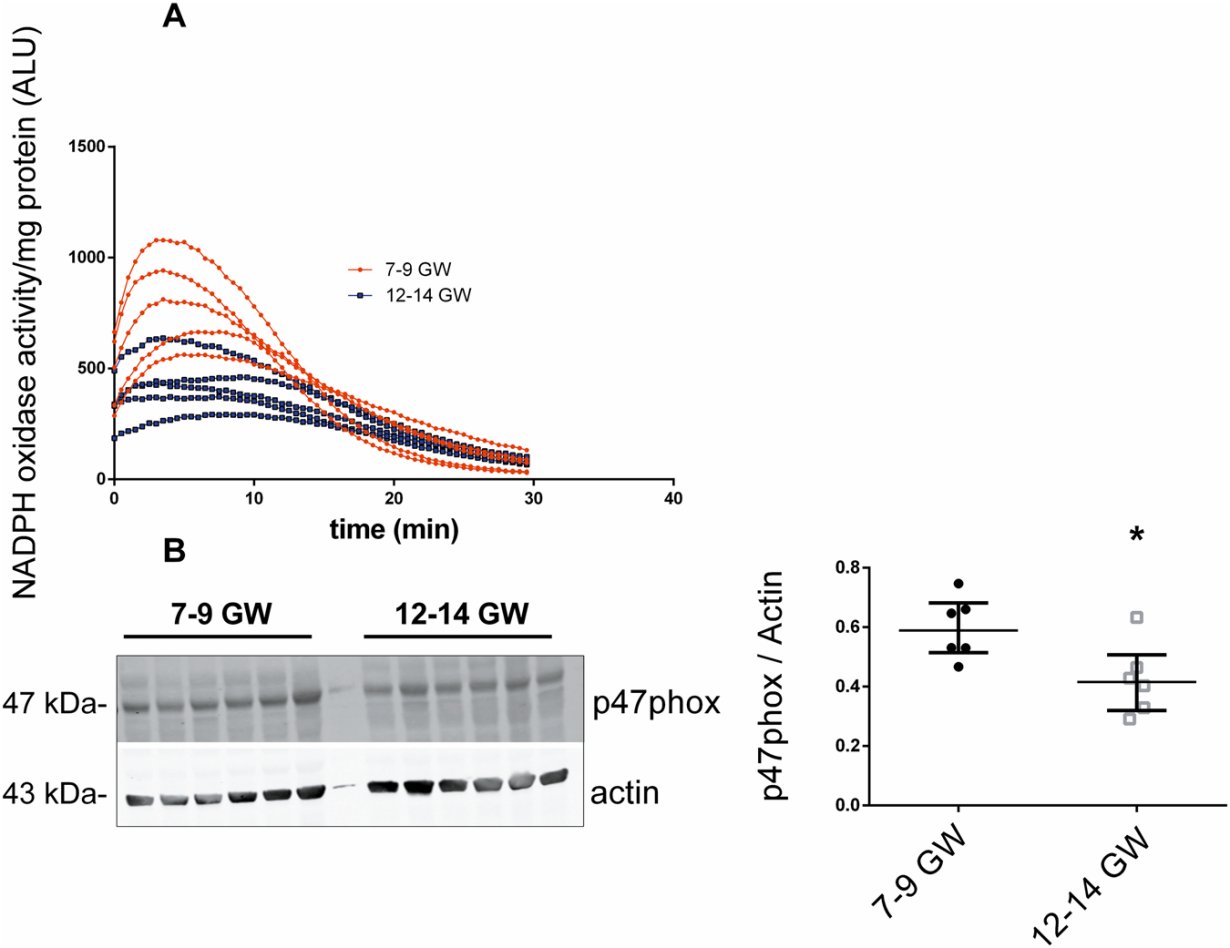
(**A**) NADPH oxidase activity was measured in first-trimester untreated chorionic villi and represented as a luminescence intensity (ALU: arbitrary light units, n = 5 different placentas) measured for 30 min. (**B**) Protein expression of p47phox subunit in chorionic villi was measured in Western blot, results are normalized to actin protein levels (n = 6 different placentas). Results are represented as median ± interquartile range. *p < 0.05. The images of bands for the target protein and actin are taken from the same blot and each image has been cropped as delineated by black dividing lines as well as adjusted for image intensity for optimal visualization.

Since Nox was found to be the major source of superoxide in first-trimester placenta, we were interested in its regulation by the environmental oxygen transition occurring in early pregnancy. Measurement of superoxide production over time showed a peak activation of Nox from 5 min after addition of NADPH that lasted about 10 min, regardless of gestational age, but a lower intensity of measured luminescence in the 12–14 GW group (Fig. 3A). After 15 min, in both groups, Nox activity returned to its original level.

**Figure 4:**
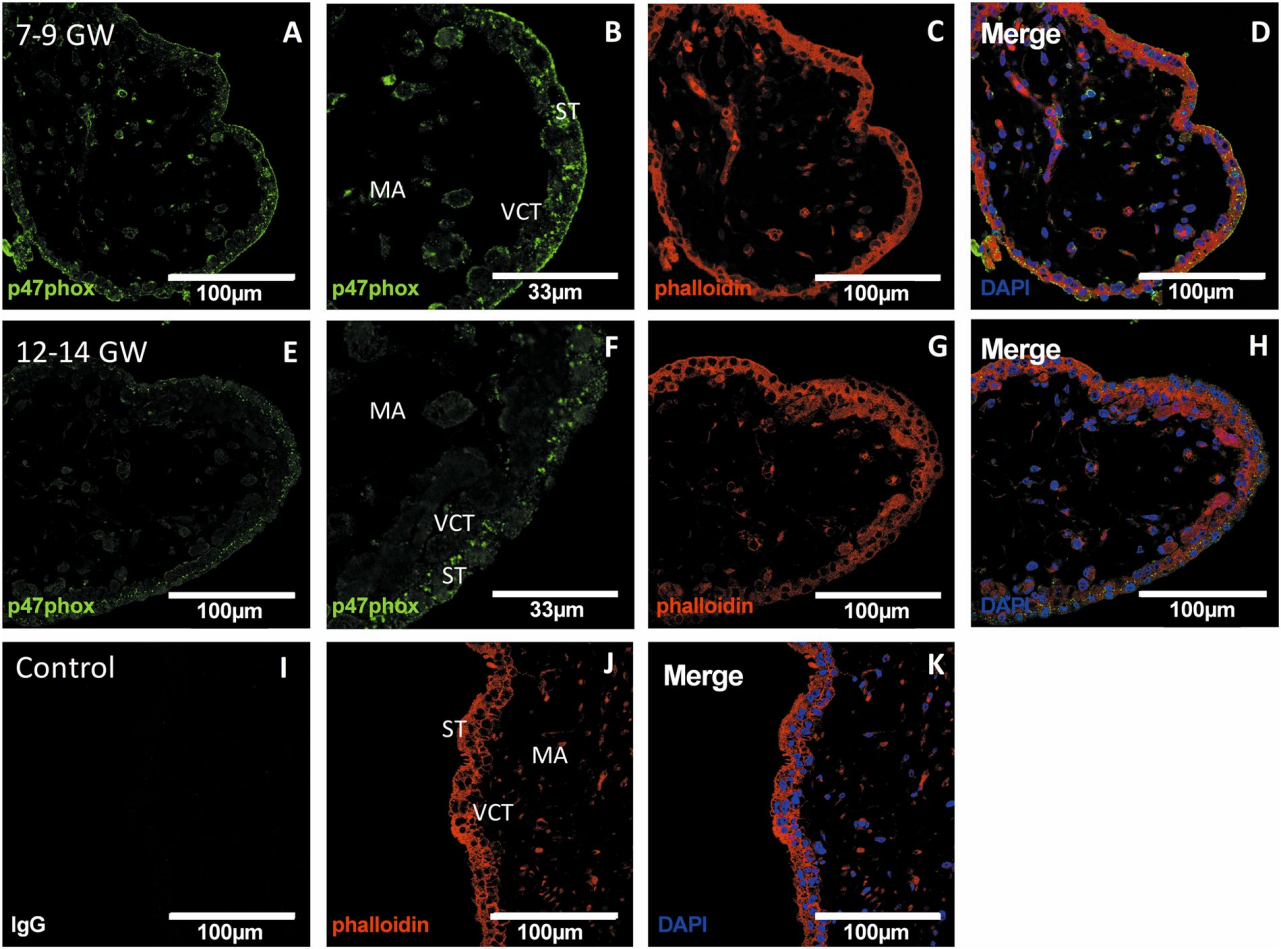
p47phox immunodetection in first-trimester chorionic villi embedded in agarose sections. (**A,B,E,F**) p47phox was stained with green 488 AlexaFluor; (**D,H,K**): nuclei were stained with DAPI; (**C,G,J**): actin-F was stained with phalloidin red 550. The IgG negative control is presented in picture (**I**), 7–9 GW chorionic villi in pictures (**A**–**D**) and 12–14 GW chorionic villi in pictures (**E**–**H**). (Confocal microscopy x400, enlargements of 7–9 and 12–14 GW chorionic villi are pictured in **B** and **F**, respectively (x1200), scale bar 100 μm or 33 μm for enlargements, n = 3 different placentas). ST: syncytiotrophoblast, VCT: villous cytotrophoblast, MA: mesenchymal axis.

To clarify Nox activity regulation, we studied the Nox subunit p47phox, because of its organizing role in the regulation of Nox activity. The protein level of the p47phox subunit in the chorionic villi was significantly higher in the 7–9 GW group (Fig. 3B). The particularity of p47phox is its ability to translocate from cytosol to cell membrane to activate Nox. Immunolocalization of p47phox showed that in chorionic villi, p47phox is mostly localized at the surface of the syncytiotrophoblast (Fig. 4A–D) before 10 GW. In contrast, in the 12–14 GW group, the majority of p47phox subunit is localized in the syncytiotrophoblast cytosol (Fig. 4E–H).

### Enzymatic antioxidant defenses are increased after the rise in oxygen pressure and mostly located in the villous cytotrophoblast (VCT)

**Figure 5:**
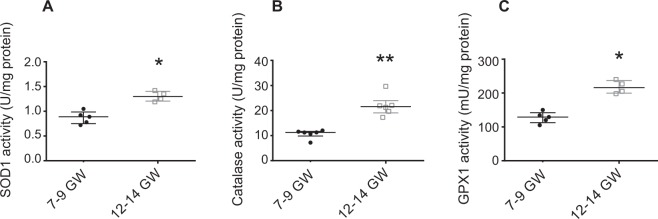
Several enzymatic antioxidant activities are measured in 7–9 vs. 12–14 GW chorionic villi from different placentas. (**A**) SOD 1 activity (7–9 GW: n = 5, 12–14 GW: n = 4); (**B**) CAT activity (7–9 GW, 12–14 GW: n = 6); (**C**) GPX 1 activity (7–9 GW: n = 5, 12–14 GW: n = 4). Activities were performed using colorimetric or spectrophotometric assay. Results are normalized to protein levels. All data are represented as median ± interquartile range. *p < 0.05, **p < 0.01.

**Figure 6:**
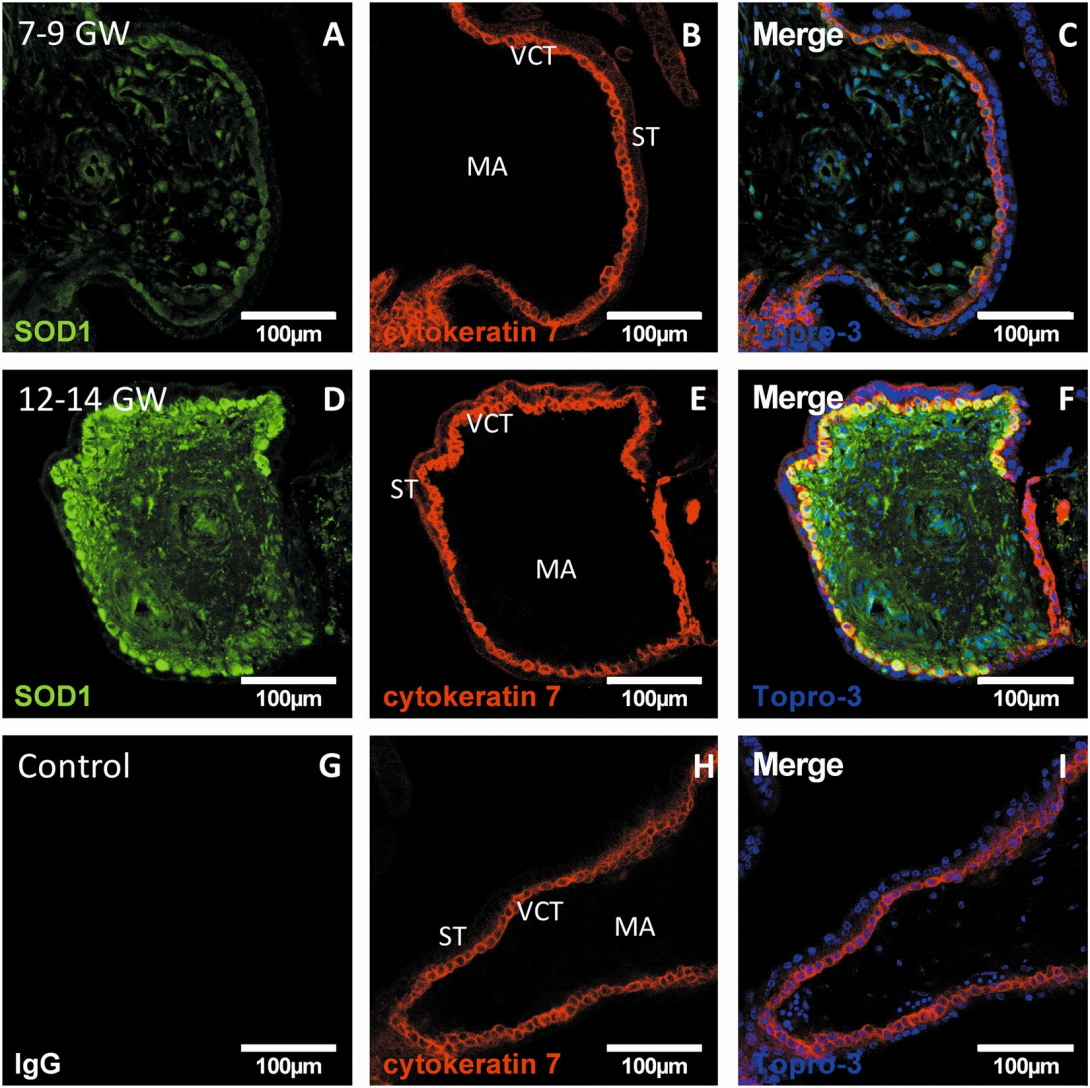
SOD1 immunodetection in first-trimester chorionic villi embedded in agarose sections. (**A**,**D**) SOD1 was stained with green 488 AlexaFluor; (**C**,**F**,**I**) nuclei were stained with TOPRO-3 and **(B,E,H**): cytokeratin 7 with red 546 AlexaFluor. IgG negative control is presented in picture (**G**), 7–9 GW chorionic villi in pictures (**A**–**C**) and 12–14 GW group chorionic villi in pictures (**D**–**F**). (Confocal microscopy x400, scale bar 100 μm, n = 3 different placentas). ST: syncytiotrophoblast, VCT: villous cytotrophoblast, MA: mesenchymal axis.

**Figure 7:**
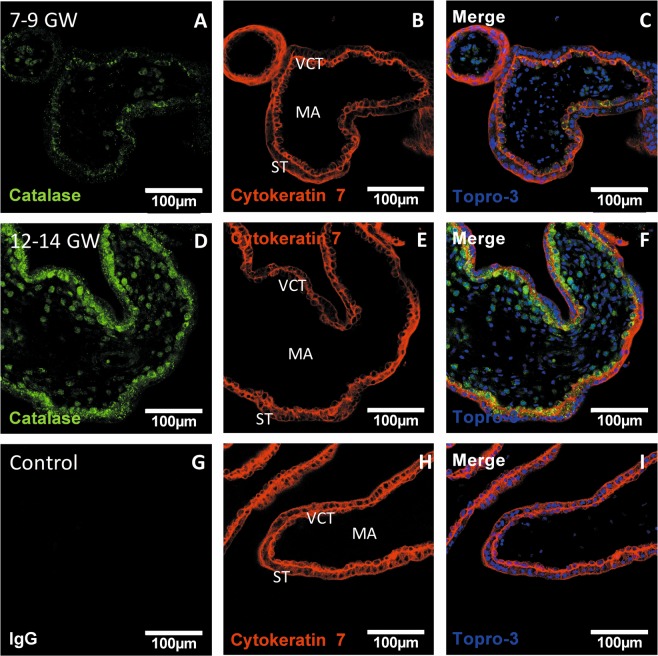
Catalase immunodetection in first-trimester chorionic villi embedded in agarose sections. (**A**,**D**) catalase was stained with green 488 AlexaFluor; **(C,F,I)**: nuclei were stained with TOPRO-3 and **(B,E,H)**: cytokeratin 7 with red 546 AlexaFluor. IgG negative control is presented in picture **(G)**, 7–9 GW group chorionic villi in pictures (**A–C**) and 12–14 GW group chorionic villi in pictures (**D**–**F**). (Confocal microscopy x400, scale bar 100 μm, n = 3 different placentas). ST: syncytiotrophoblast, VCT: villous cytotrophoblast, MA: mesenchymal axis.

To determine the adaptation of placental antioxidant defenses to the rise in oxygen pressure, we measured the activity of several antioxidant enzymes in first-trimester chorionic villi. SOD1 activity was significantly increased in the 12–14 GW chorionic villi (Fig. 5A) as was catalase (Fig. 5B) and GPX1 (Fig. 5C), compared with 7–9 GW. SOD1, which dismutes superoxide to hydrogen peroxide, and catalase, which detoxifies hydrogen peroxide to oxygen and water, are considered as first-line antioxidant defenses. Thus, the distributions of SOD1 (Fig. 6) and catalase (Fig. 7) in chorionic villi were determined. Confocal microscopy revealed a preferential localization of both enzymes in the villous cytotrophoblast, compared with the syncytiotrophoblast and mesenchymal axis (MA) (Figs 6A–F and 7A–F).

### MAPK pathways are modulated during the first trimester

MAPK pathways are well described as ROS signaling pathways^24^ and are involved in trophoblast proliferation and differentiation^16–19^. In order to determine the MAPKs pathways activities variation in our model, we used immunoblotting to analyze protein levels of phosphorylated and non-phosphorylated p38, ERK 1/2, and SAPK/JNK in 7–9 and 12–14 GW chorionic villi. The results were normalized to the level of either actin or vinculin, used here as loading controls.

**Figure 8:**
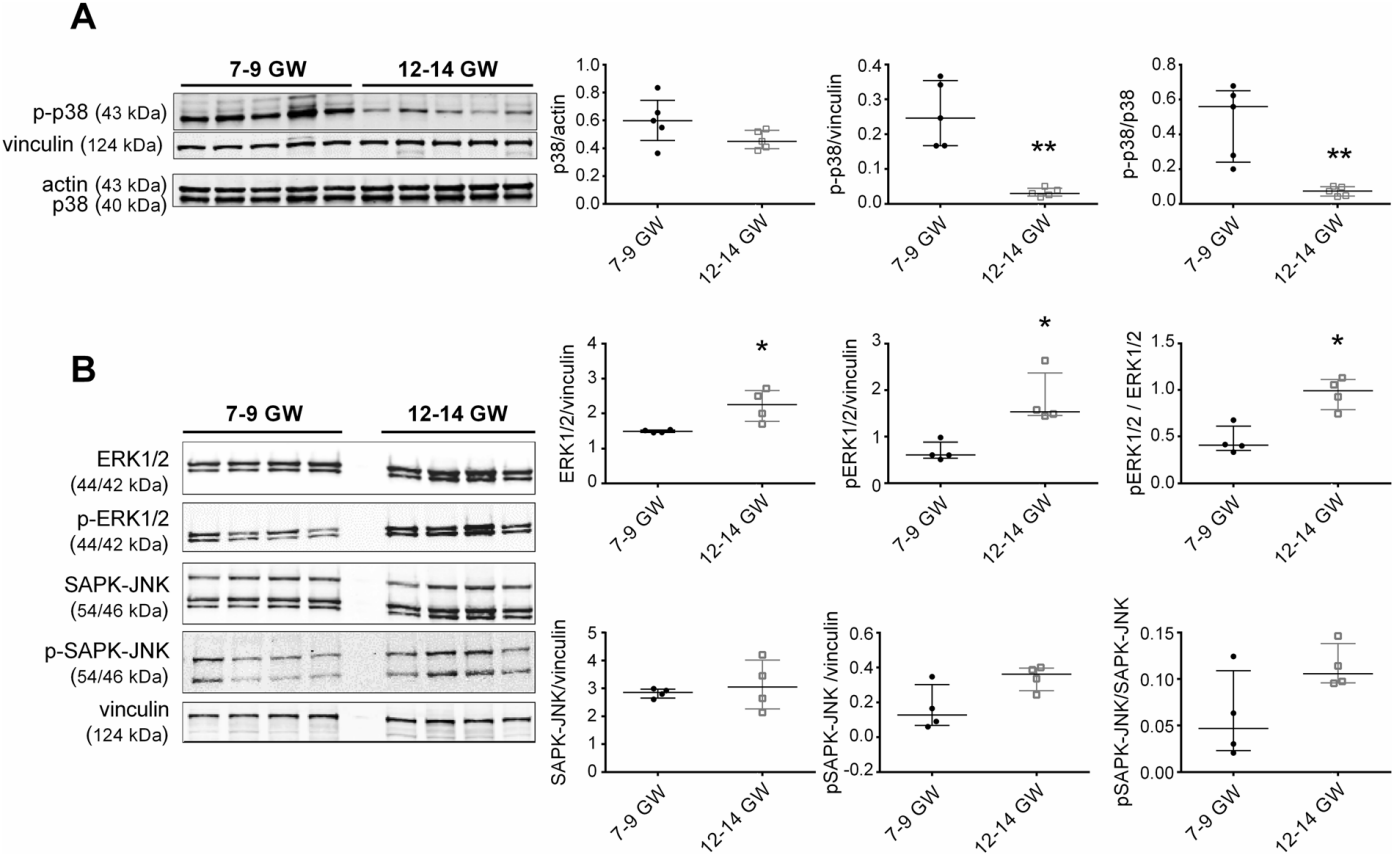
Protein lysates of first-trimester chorionic villi from different placentas (7–9 vs. 12–14 GW; n = 5 except for ERK 1/2, phospho-ERK1/2, SAPK and phospho-SAPK: n = 4) were analyzed. (**A**) Phospho-p38, p38 (**B**) phospho-ERK1/2, ERK1/2, phospho-SAPK and SAPK. Results are normalized to actin or vinculin protein expression. Data are represented as median ± interquartile range. *p < 0.05, **p < 0.01. The blot images represent the same samples which migrates in parallel on two identic gels. Representative images of blot had been cropped and delimited with black lines, as well as adjusted for image intensity for optimal visualization.

There was no difference in total p38 protein expression between the two groups, but phosphorylated p38 decreased eight-fold and therefore there was a significant seven-fold decrease in the ratio of phosphorylated p38 over total p38 (Fig. 8A). Phosphorylated and total ERK1/2 were increased about 2-fold in 12–14 GW chorionic villi. Hence, the ratio phosphorylated ERK1/2 over ERK1/2 was increased 2.5-fold (Fig. 8B). On the other hand, both phosphorylated and total SAPK/JNK protein expressions were unchanged (Fig. 8B) and no between-group differences were found in phosphorylated SAPK/JNK over total SAPK/JNK ratio.

## Discussion

In this study, we demonstrated that Nox is the principal source of placental superoxide early in pregnancy, and that Nox activity is more pronounced early in the first trimester, when the placenta is exposed to a very low oxygen pressure. For the first time in placenta, we found that this phenomenon is associated with the location of the p47phox organizer subunit, which has been found either in the cytosol or at the surface of the syncytiotrophoblast layer. The increase in NADPH oxidase activity is associated with activation of the p38 signaling pathway, whereas the ERK1/2 pathway showed decreased activation. However, the SAPK/JNK pathway activation remains unchanged. We have also observed that the activities of the other superoxide sources (mitochondrial respiratory chain, xanthine oxidase) remain unchanged during the first trimester. In parallel, we show that activities of antioxidant enzymes SOD1, catalase and GPX1 are increased after 12 GW.

Three main cellular sources of superoxide in the human placenta were explored: NADPH oxidase, the mitochondrial respiratory chain and xanthine oxidase. NADPH oxidase activity appeared to be three-fold higher in 11–13 GW than in term placenta^11^. Our study specifically focused for the first time on two separate groups (7–9 versus 12–14 GW) in the first trimester and determined the impact of oxygen rise on Nox activity and regulation. Interestingly, the activation of Nox was increased in the 7–9 GW placentas compared to the 12–14 GW placentas, which has never been described before. We also measured Nox activity in term placenta and observed a significant decrease in NADPH oxidase activity in the third-trimester placenta compared to 7–9 GW, but no difference was found compared to 12–14 GW (data not shown), suggesting a subtle regulation of superoxide production during early pregnancy and its potential involvement as a second messenger in developmental pathways. Indeed, ROS are involved in modulation of redox-sensitive pathways and especially the MAPK cascades, including ERK1/2, SAPK/JNK and p38 MAPKs^24^. These major intracellular signal transduction pathways play a key role in various cellular processes such as cell growth, differentiation, development, cell cycle, survival, and cell death^25^.

Through superoxide production, NADPH oxidase activity may impact p38 pathway activation by phosphorylation of p38^15,26^. p38 MAPK is involved in cytotrophoblast differentiation^18,27^ and proliferation^19^. Overproduction of ROS may result in cellular stress, which triggers activation of the SAPK/JNK pathway^25,28^, the core of the cell stress signaling network^29^. Here, we showed that activation of NADPH oxidase in early stages of pregnancy (<10 GW) is associated with activation of the p38 pathway, whereas the SAPK/JNK pathway remains unchanged. Hence, this suggests that the increase of Nox-dependent superoxide production does not cause cellular stress, but rather is necessary as a second messenger to activate redox-sensitive pathways. On the other hand, ERK1/2 pathways are less active when superoxide production increases. ERK1/2 is the major pathway that can be activated in trophoblasts by multiple growth factors such as epidermal growth factor (EGF) and platelet-derived growth factor (PDGF). Their receptors have been shown to be ROS targets and activate the ERK1/2 pathway^30,31^. More recent studies have highlighted the importance of regulated ROS production by NADPH oxidase in promoting EGFR-dependent signaling^32^. ERK 1/2 is involved in the regulation of the differentiation of isolated primary cytotrophoblasts into ST^27^. Nox-mediated superoxide production may play a pivotal role in mediating the activation of MAPK pathways that control trophoblast proliferation and differentiation in early pregnancy. Thus, p38 MAPK then ERK1/2 MAPK activation take turns to ensure placental differentiation: p38 is mainly activated in low oxygen conditions, but when the pressure of oxygen increases the trophoblast is more prone to differentiate into the ST, presumably mediated by the ERK1/2 pathway and protected from oxidative stress by SOD1, CAT and GPX1 enzymes, whose activities are enhanced at 12–14 GW. Indeed, the placenta develops in a low-oxygen environment that favors organogenesis of the embryo and cell proliferation in the placenta, and involvement of p38 pathway activation cannot be excluded, but remains to be tested in our model. However, the “physioxia” placental environment supplies sufficient oxygen to meet energy demands whilst also protecting cells from excess ROS concentrations that could lead to oxidative damage, particularly of the conceptus DNA^33^.

The regulation of NADPH oxidase activity appears to be under p47phox control: it is expressed at the surface of the ST when Nox activity is higher, then found in the cytosol when activity decreases, with low or absent expression at the ST apical pole. These observations suggest a p47phox translocation mechanism, which is a well-described activation mechanism of NADPH oxidase^34,35^, but has never been observed in human placenta. Despite variation in p47phox protein levels, the question of its own regulation remains. However, the main hypothesis explaining p47phox translocation from the cytosol to the membrane is the phosphorylation of its C-terminal tail on several serine residues^34,36^, which remains to be tested in our model. While the protein expression of p47phox increases at 7–9 GW, the level of gp91phox is enhanced after 10 GW (data not shown). gp91phox may have oxygen-sensing properties when combined with O_2_-sensitive K^+^ channels^37^ and its absence in carotid cells subjected to hypoxia does not alter the oxygen-sensing mechanism, whereas the lack of p47phox potentiates ventilatory and chemoreceptor responses^38^. In the placenta, O_2_-sensitive K^+^ channels have been described at the surface of ST^39^, but their association with gp91phox it not yet explored.

The antioxidant system of chorionic villi is activated by the environmental transition, which helps the human trophoblast to adapt to the rise in oxygen. We suggest that the protection against oxidative stress of the villous cytotrophoblast (VCT) cellular pool, which fuses to become ST, may be an important and efficient way that the placenta protects itself during the increase in oxygen pressure, in order to maintain the lifetime of the ST and hormone synthesis. The activation of antioxidant enzymatic defenses has been reported by others: Jauniaux *et al*. described an increase in activity of catalase and glutathione peroxidase from 9 to 16 GW, but no statistically relevant results were found concerning the total superoxide dismutase activity^3^. Raijmakers *et al*. also found increased glutathione-S-transferase activity in 11–13 GW placenta compared to term^11^. However, these studies focused mainly on placenta during the entire first trimester, often including the time window during which O_2_ pressure will change. In our work, we chose to study two distinct groups: before and after entry of the maternal blood into the intervillous space, to determine the impact of this transition on early placental development. Our results show increased antioxidant defenses in 12–14 GW placentas, which are mainly localized in villous cytotrophoblasts, suggesting a defense mechanism to maintain the VCT pool and ensure proper placental development.

A novel function of SOD1 was recently identified: SOD1 might enhance its protective role by translocating into the nucleus to protect DNA, acting as a transcription factor increasing the expression of genes involved in DNA repair^40^. This could explain the preferential localization of antioxidant enzymes as catalase and SOD1 in the VCT, in order to avoid DNA damage and apoptosis. VCT are essential to the renewal of the syncytiotrophoblast layer. Furthermore, SOD1 overexpression in cultured human VCT significantly decreases their fusion and the formation of syncytiotrophoblasts *in vitro*^41^, highlighting its significant involvement in the regulation of placental development. SOD1 function in the development process may require its activation, increasing the amount of hydrogen peroxide in the cell and explaining the later activation of catalase and glutathione peroxidase 1 in 12–14 GW placenta.

Our original findings are of interest in the pathophysiological area of placental development. Indeed, the understanding of the human placenta redox status during early pregnancy is a first step to exploring the pathogenesis of pregnancy disorders. Several teams have focused on the reliability of plasma antioxidant levels in early pregnancy as a predictive factor of pregnancy diseases^42,43^, underlining their importance in the early stage of placental development. Thus, adaptive impairment of the first-trimester placenta might lead to oxidative stress, which could result in injury to the placental tissue and other poor outcomes such as miscarriages, IUGR and preeclampsia^3,7,8,44,45^. Indeed, defective remodeling of the uterine vasculature during the first trimester leads to placental hypoxia/reoxygenation, which induces oxidative stress leading to trophoblast dysfunction. The consequence of this phenomenon is the release of excess trophoblastic factor soluble fms-like tyrosine kinase 1 (sFlt-1) into the maternal circulation, accompanied by an inflammatory response and endothelial dysfunction^46,47^. Clinical studies have associated a high ratio of sFlt-1 to placental growth factor (PlGF) with an increased risk of preeclampsia^48^. Moreover, an alteration in the sFlt-1/PlGF ratio occurs several weeks before the onset of clinical signs and is correlated with the severity of the disease^49^.

Alteration of Nox activity may be implicated in the pathogenesis of preeclampsia, especially in inflammatory and neovascularization processes^50,51^. An increase in the Nox1 isoform has been described in preeclamptic term placentas^52^ localized in the ST and stromal cells. More and more studies highlight the possible relationship between Nox, ROS signaling pathways and the onset of preeclampsia via the inflammatory response due to the activation of the nuclear factor-kappa B (NF-κB) pathway^50^ or the synthesis of sFlt-1 by the extravillous cytotrophoblast cell line HTR8/SVNeo^53^. The novel data described in this study, on the regulation of placental Nox during various stages of first-trimester pregnancy, appear essential to a better understanding of the molecular mechanisms of such pathology^54^.

The first validation of NADPH oxidase as a therapeutic target was introduced with Nox2- and then Nox1-deficient mice^55^. Further, pharmacological inhibition strategies and genetic manipulations in animal models (mice in which one of the Nox genes is inactivated or overexpressed) have also been applied. The identification of placental Nox as a major producer of superoxide in the early stage of pregnancy, and the understanding of the mechanisms involved in its activity and function, are now required to study placental Nox as a therapeutic target in placental diseases.

## Conclusion

First-trimester placenta is exposed to an increase in oxygen levels between 10 and 12 GW and needs therefore to adapt its redox balance to ensure the maintenance of pregnancy: the main antioxidant enzymes SOD1, catalase, and GPX1 are activated in 12–14 GW placenta, with no variation of the SAPK/JNK pathway during the first trimester. In this study, we demonstrate for the first time that Nox is the main placental source of superoxide during the first trimester and that Nox activity is increased in 7–9 GW placenta - with the p47phox organizer subunit located on the cell membrane of the ST - associated with the activation of the p38 MAPK pathway. These findings may highlight the leading role of Nox in placental functions and differentiation through redox-sensitive pathways and suggest its involvement in the pathogenesis of preeclampsia.

## Data Availability

All data are available on request.
